# Pharmacogenomic–pharmacokinetic study of selective estrogen-receptor modulators with intra-patient dose escalation in breast cancer

**DOI:** 10.1007/s12282-019-00952-9

**Published:** 2019-02-07

**Authors:** Hiroshi Ishiguro, Shinji Ohno, Yutaka Yamamoto, Shintaro Takao, Nobuaki Sato, Tomomi Fujisawa, Takayuki Kadoya, Katsumasa Kuroi, Hiroko Bando, Yasufumi Teramura, Hiroji Iwata, Shiro Tanaka, Masakazu Toi

**Affiliations:** 1 Department of Medical Oncology, International University of Health and Welfare Hospital, 537-3 Iguchi, Nasushiobara, Tochigi 329-2763 Japan; 2 Center of Breast Oncology, The Cancer Institute Hospital of JFCR, Tokyo, Japan; 3 Department of Molecular-Targeting Therapy for Breast Cancer, Kumamoto University Hospital, Kumamoto, Japan; 4 Department of Breast Surgery, Hyogo Cancer Center, Akashi, Japan; 5 Department of Breast Surgery, Niigata Cancer Center Hospital, Niigata, Japan; 6 Department of Breast Oncology, Gunma Prefectural Cancer Center, Ohta, Japan; 7 Department of Surgical Oncology, Hiroshima University Hospital, Hiroshima, Japan; 8 Department of Breast Surgery, Tokyo Metropolitan Cancer and Infectious Diseases Center Komagome Hospital, Tokyo, Japan; 9 Breast and Endocrine Surgery, Faculty of Medicine, University of Tsukuba, Tsukuba, Japan; 10 Department of Surgery, Hikone Municipal Hospital, Hikone, Japan; 11 Department of Breast Oncology, Aichi Cancer Center Hospital, Nagoya, Japan; 12 Department of Pharmacoepidemiology, Graduate School of Medicine and Public Health, Kyoto University, Kyoto, Japan; 13 Department of Breast Surgery, Graduate School of Medicine Kyoto University, Kyoto, Japan; 14 Japan Breast Cancer Research Group (JBCRG), Tokyo, Japan

**Keywords:** Tamoxifen, Toremifene, CYP2D6, Pharmacokinetics, Breast cancer

## Abstract

**Background:**

An association between *CYP2D6* polymorphisms and tamoxifen (TAM) efficacy has not been confirmed, partly due to unreliable prediction of active metabolite exposure solely by CYP2D6 activity. The efficacy of TAM dose escalation appears limited in poor TAM metabolizers. Since the chlorine atom on the side chain of toremifene (TOR) prevents 4-hydroxylation by CYP2D6, its contribution to active conversion of TOR is minor. We examined the role of TOR and its dose escalation among poor TAM metabolizers.

**Methods:**

The pharmacokinetics (PK) and pharmacogenomics (PGx) of TAM and TOR were studied. Correlation between PK and CYP2D6 inhibitor use, smoking status, and PGx were examined by regression analysis. For patients showing low endoxifen levels, an intra-patient dose escalation of TOR was conducted, and TOR was increased from 40 to 120 mg for ≥ 24 weeks with PK sampling. Total activity was calculated as the sum of the concentration of each active metabolite adjusted by their respective in vitro activities.

**Results:**

Fifty and 11 of the 273 participating patients had endoxifen levels < 15 and < 7.5 ng/mL, respectively. The *CYP2D6* genotype was the major determinant for TAM activity (p < 0.01). Smoking status (*p* = 0.07) and the CYP2C19 phenotype (*p* = 0.07), but not the *CYP2D6* genotype (*p* = 0.61), showed marginally significant effects on TOR activity. TOR activity increased significantly with dose escalation, even among poor TAM metabolizers, and was maintained for ≥ 24 weeks.

**Conclusion:**

TOR might be a valid alternative to TAM in patients predicted to be poor TAM metabolizers.

**Electronic supplementary material:**

The online version of this article (10.1007/s12282-019-00952-9) contains supplementary material, which is available to authorized users.

## Introduction

The relationship between *CYP2D6* polymorphisms and the therapeutic effects of tamoxifen (TAM) has been studied for a decade, but the results are contradictory and inconclusive. Regarding the question raised in the article [1], “How predictive is the *CYP2D6* genotype for endoxifen and 4-hydroxy (4OH)-TAM levels?” the data show that this genotype can be used to compare populations, but there are large overlaps in pharmacokinetics/pharmacodynamics (PK/PD) of individual patients belonging to different genotype groups. There are several possible explanations for the variations in the literature [1–3]. First, the frequencies of the *CYP2D6* alleles differ between Asian and Caucasian populations, resulting in inconsistencies in the effects of TAM depending on the study location. Second, the methodology, classification, and scoring used for *CYP2D6* polymorphisms differ among studies. Third, because many other enzymes are involved in the metabolism, it is impossible to explain the differences in the PK/PD solely using *CYP2D6* polymorphisms. Association between tamoxifen PK and *CYP2C19* [4] or *ABCC2* (*rs3740065*) [5] polymorphism has been reported. Fourth, concomitant usage of CYP2D6 inhibitors has a greater impact on PK than genetic polymorphisms. Fifth, poor compliance among extensive metabolizers (EMs) is expected. Sixth, retrospective studies with samples chosen based on availability and representing a small proportion of the overall population are not representative of the entire population and are statistically underpowered. Finally, genotyping from formalin-fixed, paraffin-embedded specimens may not be as accurate as that from the DNA of blood lymphocytes, because somatic deletion at the *CYP2D6* chromosomal locus is relatively common in breast cancer. Unless these issues are addressed, conducting similar studies will not determine the influence of *CYP2D6* polymorphisms on the therapeutic effects of TAM.

Even among genetically determined EMs, large inter-patient variability exists in the plasma concentrations of active metabolites, endoxifen, and 4OH-TAM. A significant proportion of EMs have blood levels of active metabolites close to those of patients who are genotypically homozygous for unstable enzymes with reduced activity alleles [6, 7], and some even have levels close to those of patients who are genotypically homozygous for nonfunctional alleles [8]. In addition, patients who concurrently take selective serotonin reuptake inhibitors such as paroxetine achieve similar active metabolite concentrations to those who are genotypically homozygous for nonfunctional alleles [8]. Although Wu et al. indicated that endoxifen levels in the blood can be estimated from information such as age, race, and genotype [9], direct determination of drug concentrations is considered a more suitable approach, as is common practice for anticonvulsants and immunosuppressants.

Recent clinical pharmacology studies have indicated that simply increasing the dose of TAM may not overcome the genetically reduced enzymatic activity of CYP2D6 [6, 10]. Therefore, we chose a different strategy using another selective estrogen-receptor modulator that is metabolically independent of CYP2D6, toremifene (TOR), and conducted a clinical pharmacology study to confirm the effectiveness of our strategy. We enrolled breast cancer patients with low endoxifen levels on 20 mg of TAM into our intra-patient dose-escalation study of TOR (initial dose, 40 mg; escalated dose, 120 mg), for which the contribution of CYP2D6 to its bioactivation seemed lower than that for TAM [11], to investigate the intra-patient differences in the total activity of TOR calculated by the blood concentrations of the active metabolites between the 40- and 120-mg doses.

## Patients and methods

### Study design and patient population

The present study includes the screening study “Pharmacokinetics–pharmacogenomics study in breast cancer endocrine treatment” and an intra-patient dose-escalation clinical pharmacology study for TOR, “PGx–PK-based clinical pharmacology study of anti-estrogens.”

For the screening study, we enrolled any breast cancer patients taking either TAM or TOR who maintained good (85% or above) compliance for at least 12 weeks, with adequate organ function. Patients who needed regular use of CYP2D6 inhibitors such as paroxetine, sertraline, amiodarone, or metoclopramide were excluded.

The eligibility criteria for the intra-patient dose-escalation clinical pharmacology study for TOR included breast cancer patients who had participated in the screening study and were found to have very low-active metabolite levels as defined by endoxifen levels of < 7 (about a half of endoxifen concentration for extensive metabolizers) or low endoxifen levels of < 15 ng/ml (about a quarter of endoxifen concentration for extensive metabolizers) with one of the following three features such as no hot flushes, at least one null genotype for CYP2D6 (*3, 4, 5, 14, 18, 21, 44), or two alleles of a low-activity genotype for CYP2D6 (*9 or 10). Patients taking CYP2D6 inhibitors or who had a history of thromboembolism, severe comorbidities, or inadequate organ function were excluded. Written informed consent was obtained from all the participants included in the study. The patient enrolment period for the screening study started on February 1, 2009 and ended on July 31, 2012. The enrolment period for the intra-patient dose-escalation clinical pharmacology study for TOR started on November 1, 2009 and ended on November 30, 2013.

### Pharmacogenomics

Pharmacogenomics (PGx) analysis for *CYP2D6, CYP2C19*, and *ABCC2 (MRP2)* was conducted by FALCO Biosystems (Kyoto, Japan). Genomic DNA was extracted from the whole blood of patients using a QIAamp DNA Blood Mini Kit (QIAGEN GmbH, Hilden, Germany). CYP2D6*3, *4, *9, and *10 were genotyped by an allele-specific PCR assay followed by agarose gel electrophoresis. CYP2D6*5 was genotyped by a long-range PCR assay followed by agarose gel electrophoresis as described previously [9, 10]. CYP2D6*14, *18, *21, and *44, and *MRP2 (rs3740065)* were genotyped by Sanger sequencing using the 3103xl Genetic Analyzer, and Sequencing Analysis Software (v5.3.1; Life Technologies Corporation, Carlsbad, CA, USA) was used for the analysis. CYP2C19*2 and *3 were genotyped by the TaqMan Drug Metabolism Assay (Life Technologies Corporation) for *2 (C__25986767_70) and *3 (C__27861809_10) using the 7900HT Sequence Detection System, with analysis by the SDS Software (v2.3; Life Technologies Corporation).

These genotypes are classified into six subtypes, as follows: type 1, homozygous for two null genotypes (*3, *4, *5, *14, *18, *21, *44); type 2, *null*/*wild*; type 3, homozygous for two low-active genotypes (*9 or *10), *low*/*low*; type 4, *null*/*low*; type 5, *low*/*wild*; type 6, *wild*/*wild*. The *CYP2C19* genotypes are classified into: EM, homozygous for *wild type*; poor metabolizer (PM), homozygous for mutant allele (*2, *3); intermediate metabolizer (IM), heterozygous (*2/*wild* or *3/*wild*).

### Pharmacokinetics

Blood samples for PK analysis were obtained from patients who had good compliance with taking medications 2–4 h after taking anti-estrogens. For the intra-patient dose-escalation clinical pharmacology study for TOR, blood sampling was performed after administering TOR 40 mg for at least 4 weeks, after which the dose was increased to 120 mg. PK sampling was performed twice: the first sampling was performed after at least 4 weeks when PK reached a steady state; 120 mg TOR was administered for another 20–28 weeks after which the second sampling was performed to assess PK stability over time (Figure S1). The plasma was analyzed for the concentrations of TAM and TOR, as well as the 4OH-, *N*-desmethyl (NDM-), and 4-hydroxy-*N*-desmethyl (4OH-NDM-) metabolites of TAM and TOR, respectively, using LC–MS/MS by NAC Co., Ltd., under the “Guidance for Industry: Bioanalytical Method Validation” issued in May 2001 by the US Department of Health and Human Services, the Food and Drug Administration, the Center for Drug Evaluation and Research, and the Center for Veterinary Medicine. Standard curves were prepared in the concentration range of 20–2000 ng/mL for TOR, 4OH-TOR, TAM, and 4OH-TAM, and 0.20–50 ng/mL for NDM-TOR, 4OH-NDM-TOR, and NDM-TAM, and 0.50–50 ng/mL for endoxifen. The interday variability and intraday variability for all compounds are within 15%.

The total activity of TAM was calculated as 1.0 × TAM + 0.655 × NDM-TAM + 116 × 4OH-TAM + 46.3 × endoxifen, and the total activity of TOR was calculated as 1.0 × TOR + 0.764 × NDM-TOR + 98.0 × 4OH-TOR + 91.9 × 4OH-NDM-TOR [11].

### Adverse events and intra-patient dose escalation

We obtained data on compliance and the grade of hot flush as defined by the Common Terminology Criteria for Adverse Events v3.0.

Patients were started on 40 mg of TOR. After 4 weeks, the dose of TOR was increased to 120 mg, and continued as long as adverse events for which a causal relationship with TOR could not be ruled out did not exceed grade 1.

### Data management and statistical methodology

Because no previous clinical trials have used the total activity as an endpoint, the patient sample size for the intra-patient dose-escalation clinical pharmacology study for TOR was tentatively calculated as 30 to show that the total activity of TOR 120 mg is statistically higher than that of TOR 40 mg with a 95% confidence interval, assuming that 20% of the patients will not complete the entire study period. Sample sizes for the screening study were set at 200 and 100 for TAM- and TOR-treated patients, respectively, to attain approximately 60 poor metabolizers (PMs) for TAM, with twice as many candidates for the intra-patient dose-escalation clinical pharmacology study for TOR. A paired *t* test or McNemar’s test was used to compare the adverse events between different TOR doses/timing. The *p* value was not calculated if there were no events. Paired or unpaired *t* tests were used to compare the TOR metabolite concentration or the total activity between different doses/timing of TOR.

## Results

### Patients’ background

**Fig. 1:**
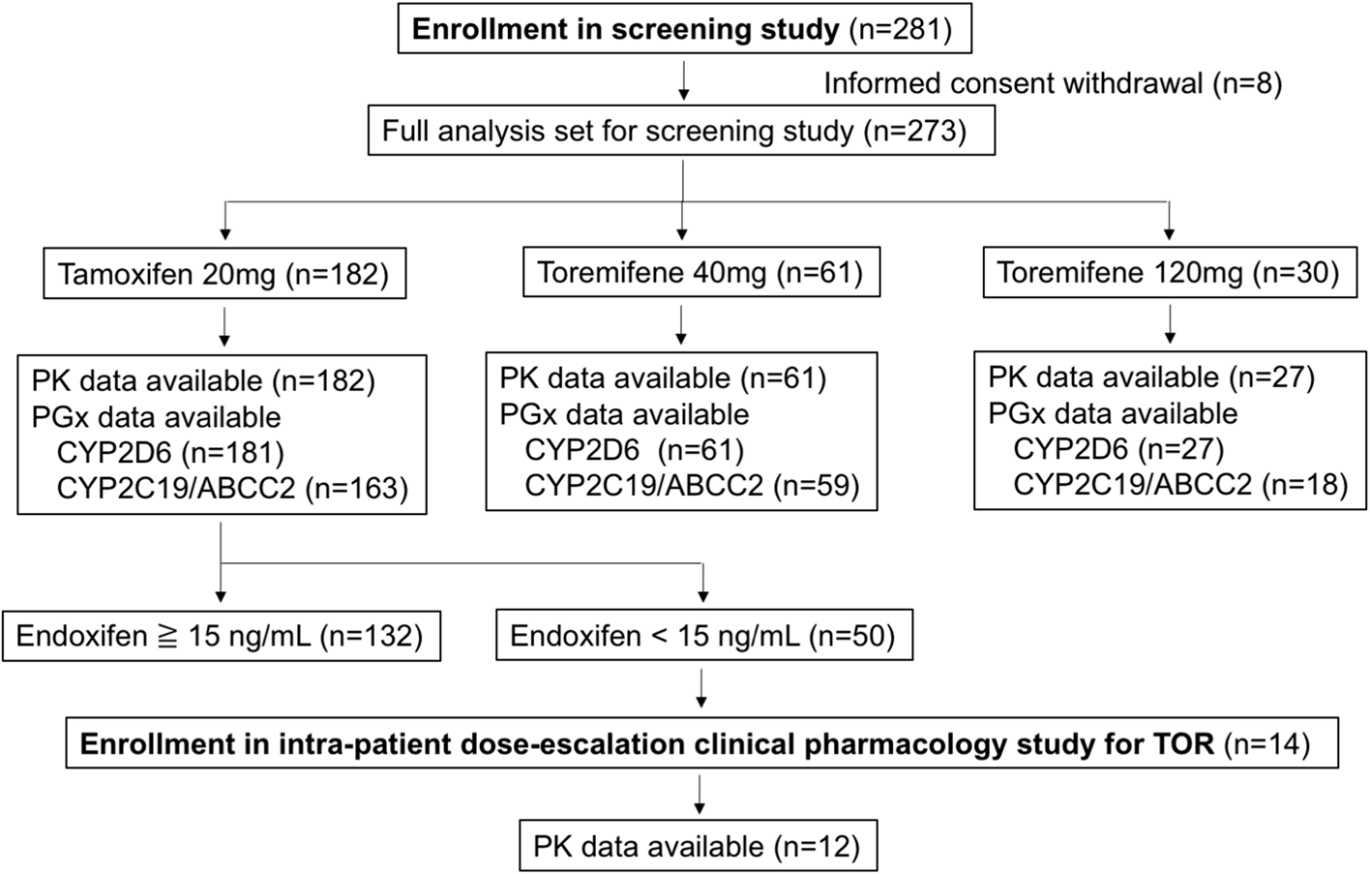
Diagram showing clinical trial enrollment and study schema

**Fig. 2:**
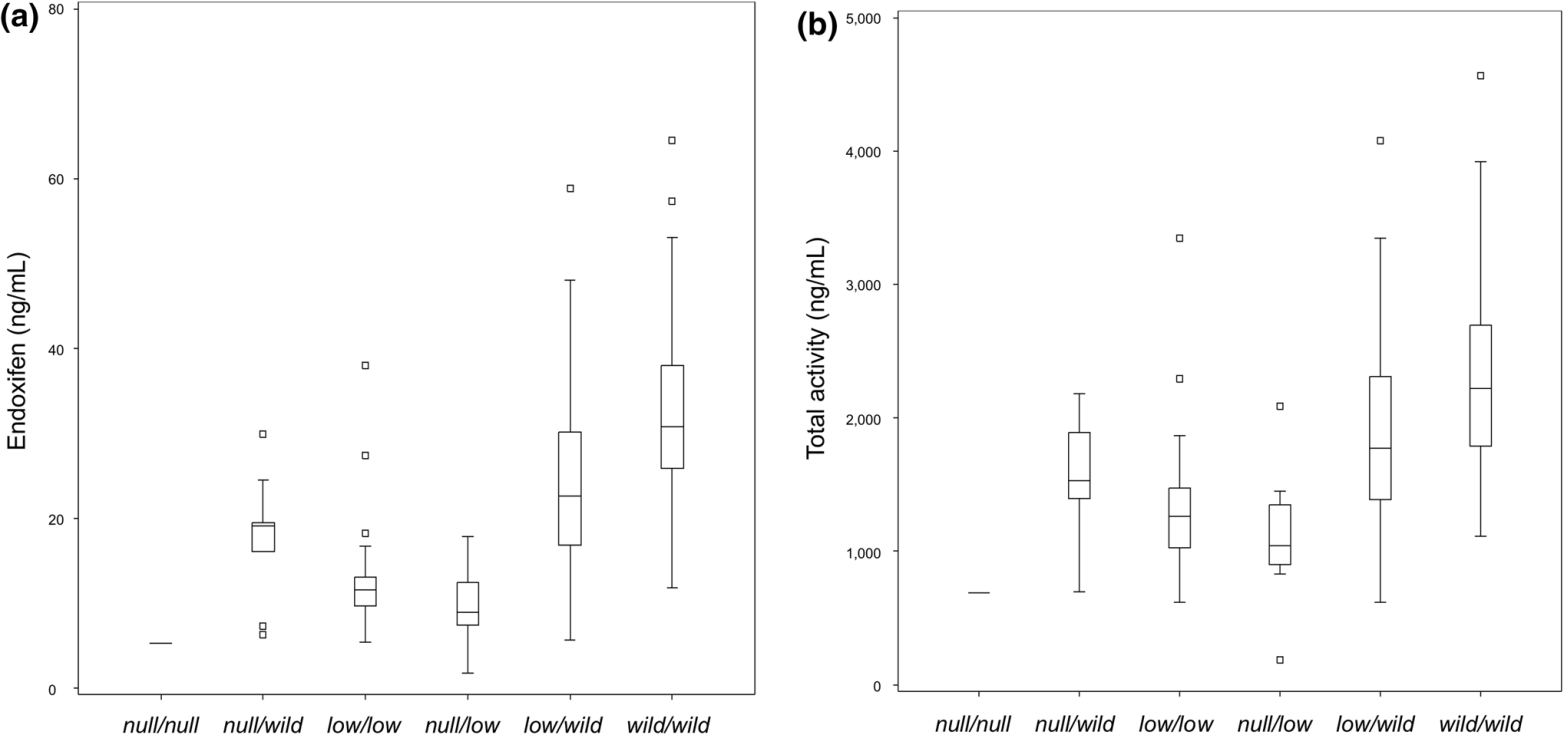
Distribution of endoxifen (**a**) and total activity (**b**) of patients in the screening study (SC)

Figure 1 illustrates the study schema. The full analysis set for the screening study included 273 patients. Among them, 182 patients were on TAM, and 61 and 30 patients were on TOR 40 mg and 120 mg, respectively. PK sampling was not conducted for three patients on TOR 120 mg. Table 1 shows the background of these patients. A lower grade and frequency of hot flushes was observed in patients taking TOR 120 mg than in the patients taking TAM 20 mg or TOR 40 mg. Low percentages of the patients were current smokers (5.5%) or users of CYP inhibitors (10.6%). Only one patient used strong CYP2D6 inhibitor (paroxetine). Figure 2a, b illustrates the relationship between CYP2D6 phenotype and endoxifen concentration or the total activity of TAM. Comparably, low concentrations of endoxifen and total activity were found in the patients with *CYP2D6 low*/*low* and *CYP2D6 null*/*low genotype*. In addition, significant proportions of patients with *low*/*wild* and *wild*/*wild* genotypes had low concentrations of endoxifen and total activity, which are similar results to those for the patients with *CYP2D6 low*/*low* and *CYP2D6 null*/*low genotypes*.

**Table 1:** Background of the 273 patients in the screening study

	Mean	SD	Median	Min	Max
Age (years)	52.5	10.7	50.0	27.0	84.0
Weight (kg)	53.5	8.4	52.9	30.0	90.0
Height (cm)	156.6	5.7	156.5	140.0	173.0
BSA (m^2^)	1.52	0.12	1.51	1.11	1.94
Anti-estrogen/dose					
TAM/20 mg	182	66.7%			
TOR/40 mg	61	22.3%			
TOR/120 mg	30	11.0%			
Hot flush grade					
0	164	60.1%	273		
1	81	29.7%			
2	27	9.9%			
3	1	0.4%			
Hot flush grade					
0	105	56.8%			
TAM 20 mg					
1	58	31.4%			
2	21	11.4%			
3	1	0.5%			
Hot flush grade					
0	35	57.4%			
TOR 40 mg					
1	20	32.8%			
2	6	9.8%			
3	0	0.0%			
Hot flush grade					
0	24	88.9%			
TOR 120 mg					
1	3	11.1%			
2	0	0.0%			
3	0	0.0%			
Smoking					
Yes	15	5.5%			
No	254	93.0%			
Unknown	4	1.5%			
CYP inhibitor use					
2D6	3	1.1%			
3A4	12	4.4%			
Both	14	5.1%			
None	244	89.4%			

Among the 182 patients on TAM, 50 patients had endoxifen levels lower than 15 ng/mL. Among these patients, 14 PMs of TAM as defined by endoxifen concentration and *CYP2D6* genotype were enrolled in the intra-patient dose-escalation clinical pharmacology study for TOR. Table 2 shows the background of these patients. Among the 14 patients enrolled, 12 patients completed the study, and PK data were obtained for these patients. No patients had the *wild*/*wild* genotype for *CYP2D6*, and 50% of these patients were homozygous for the low phenotype. The mean and median concentrations for endoxifen in the patients on 20 mg of TAM were 9.7 and 10.4 ng/mL, respectively, in contrast with 23.3 and 22.6 ng/mL for the entire population, respectively.

**Table 2:** Background of the 14 patients in the intra-patient dose-escalation clinical pharmacology study for TOR

	Mean	SD	Median	Min	Max
Age (years)	50.1	7.9	47.0	43.0	68.0
Height (cm)	159.7	5.1	159.8	151.0	167.0
Performance status					
0	14	100.0%			
1–2	0.0	0.0%			
Endoxifen on TAM 20 mg	9.7	3.1	10.4	1.8	12.6
CYP2D6 phenotype					
1 (null/null)	0	0.0%			
2 (null/wt)	0	0.0%			
3 (low/low)	7	50.0%			
4 (null/low)	5	35.7%			
5 (low/wt)	2	14.3%			
6 (wt/wt)	0	0.0%			
Hot flush grade on TAM 20 mg					
0	8	57.1%			
1	4	28.6%			
2	2	14.3%			
CYP inhibitor use					
2D6	0	0.0%			
3A4	1	7.1%			
Both	1	7.1%			
None	12	85.7%			

### Pharmacokinetics

Table S1 shows a summary of the concentrations of TAM, TOR, and their metabolites. The mean and median endoxifen concentrations were 23.5 and 22.8 ng/mL, respectively, with large intra-patient variability (1.8–64.5 ng/mL) for patients taking TAM 20 mg a day. The mean and median concentrations of the other active metabolite of TAM, 4OH-TAM, were only 3.6 and 3.3 ng/mL, respectively.

The mean concentrations of the 4OH metabolites of TOR, 4OH-TOR and 4OH-NDM-TOR, in the patients taking TOR 40 mg were 8.1 and 15.4 ng/mL, and in those taking TOR 120 mg were 16.0 and 32.6 ng/mL, respectively, compared to the concentrations of the parent compound, TOR, in those taking TOR 40 mg (732 ng/mL) and those taking TOR 120 mg (1957.5 ng/mL), indicating a lower contribution of the 4OH-metabolizing enzyme, CYP2D6, for TOR than for TAM.

### Multiple regression analysis of TAM and TOR pharmacokinetics

As shown in Table 3, the CYP2D6 phenotype was significantly associated with the total activity of TAM but not for TOR. On the other hand, in addition to the TOR dose, current smoking status and CYP2C19 phenotype seem to be associated with TOR activity.

**Table 3:** Associations between the total activity of TAM or TOR and gene polymorphisms

	Total activity of TAM (*N* = 159)				Total activity of TOR (*N* = 77)			
	Mean	95% CI		*p*	Mean	95% CI		*p*
Intercept	2063.3	1468.9	2657.7	–	10315.0	7924.2	12706.0	–
Dose of TOR								
40 mg	–	–	–	–	−4903.1	−6052.7	−3753.4	< 0.01
120 mg	–	–	–	–	Ref	–	–	–
CYP2C19 phenotype								
EM	−79.7	−441.6	282.3	0.90	979.6	−584.3	2543.5	0.07
Hetero	−75.9	−413.1	261.4	–	−171.9	−1679.6	1335.7	–
PM	Ref	–	–	–	Ref	–	–	–
CYP2D6 phenotype								
1 (null/null)	−1569.1	−2874.5	−263.6	< 0.01	No patient	–	–	0.61
2 (null/wt)	−768.0	−1269.3	−266.6	–	−593.5	−2523.4	1336.5	–
3 (low/low)	−1034.8	−1358.7	−710.9	–	−685.9	−2067.5	695.7	–
4 (null/low)	−1232.2	−1661.4	−803.1	–	−713.2	−3644.0	2217.5	–
5 (low/wt)	−442.7	−682.9	−202.4	–	303.4	−773.8	1380.7	–
6 (wt/wt)	Ref	–	–	–	Ref	–	–	–
ABCC2 genotype								
mut/mut	102.9	−214.2	420.0	0.33	716.8	−583.2	2016.9	0.46
mut/wt	171.0	−53.8	395.7	–	581.8	−480.6	1644.1	
wt/wt	Ref	–	–	–	Ref	–	–	–
Current smoking status	275.1	−199.3	749.4	0.25	−2265.1	−4743.8	213.6	0.07
Concurrent strong 3A4 or 2D6 inhibitor use	−786.5	−2089.5	516.4	0.23	1187.1	−2733.5	5107.8	0.55

### Intra-patient dose escalation and adverse events

With an increase in the dose of TOR, only a few patients developed hot flushes and GOT/GPT elevation (Table S2). Body weight remained stable.

**Fig. 3:**
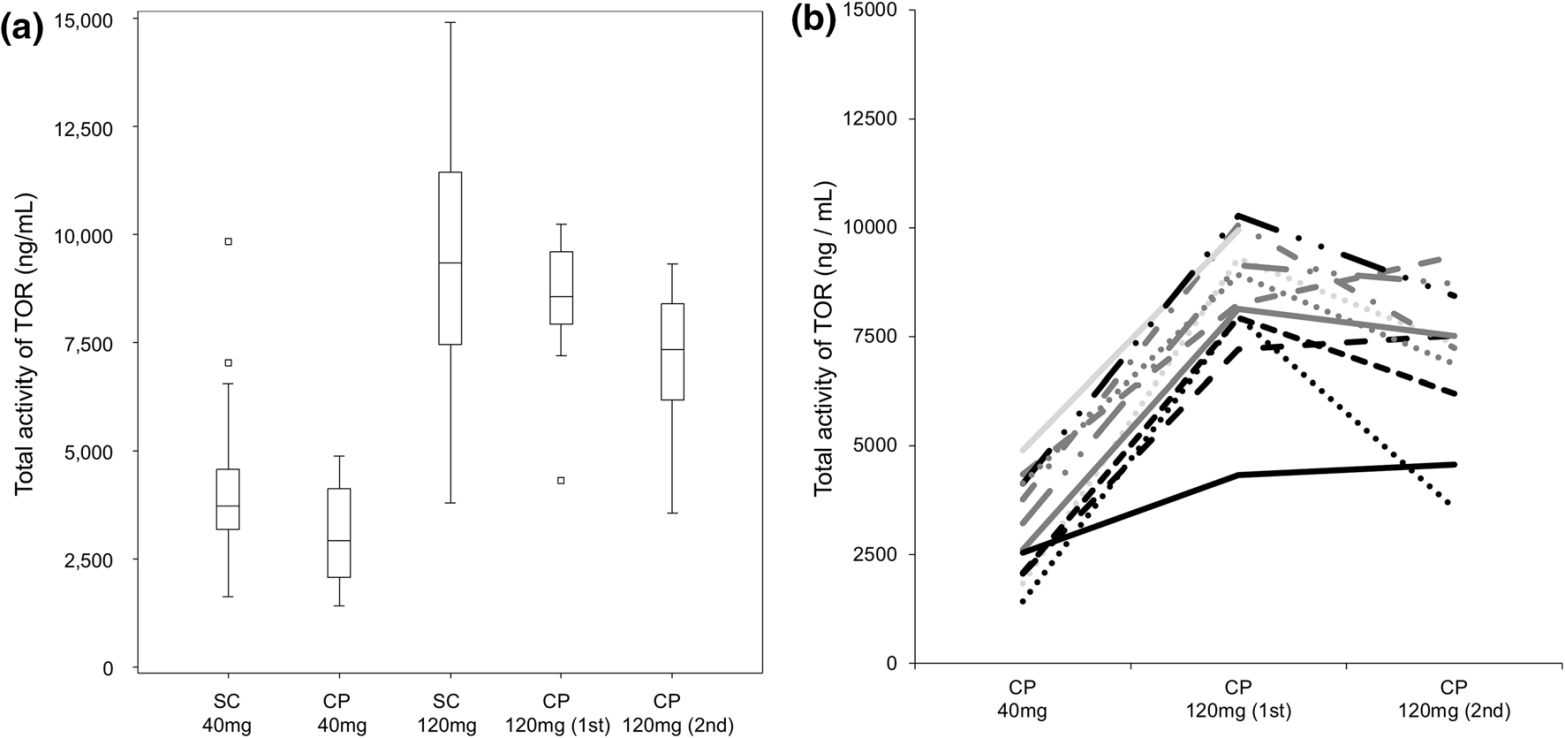
Distribution of the total activity of patients in the screening study (SC) and the intra-patient dose-escalation clinical pharmacology study for TOR (CP 1st and 2nd) (**a**) and individual patients’ levels during dose escalation and maintenance in the intra-patients dose-escalation clinical pharmacology study for TOR (**b**)

Figure 3a, b shows the total activity of TOR in the screening study and during intra-patient dose escalation, respectively. Almost all the metabolites increased in concentration at least twofold after increasing the dose from 40 to 120 mg (data not shown). The total activity also increased by at least twofold (*p* < 0.01, Fig. 3a, b). By continuing the same dose of TOR for up to 6 months, the concentration of TOR and its metabolites, as well as the total activity of TOR, remained constant, although there was a trend for decreased total activity after 24 week continuation of high-dose TOR (Fig. 3a, b).

## Discussion

This is one of the few studies in which data on both concomitant use of CYP inhibitors (both 2D6 and 3A4) and an extensive list of genetic polymorphisms within *CYP2D6, CYP2C19*, and *ABCC2* were analyzed together, with data obtained from a non-Caucasian population. The results of the screening component of the study indicated that the CYP2D6 phenotype is the major determinant for TAM activity but not for TOR. Conversely, significant proportions of patients with *CYP2D6 low*/*wild* and *wild*/*wild* genotypes who were not using concurrent CYP2D6 inhibitors had lower endoxifen levels comparable to those of patients with the *low*/*low* and *null*/*low genotypes*, which may suggest the importance of therapeutic drug monitoring to optimize drug exposure for individual patients. Furthermore, the intra-patient dose-escalation part of the study showed that switching to TOR 40 mg and increasing the dose to 120 mg is a valid option to overcome TAM underexposure for PMs of TAM.

There are several limitations in this study. First, the only endocrine-related adverse event evaluated was hot flushes, and other symptoms reportedly related to TAM efficacy such as night sweats, musculoskeletal adverse events, and vulvovaginal symptoms were not evaluated [12–14]. Frequency and severity of adverse events were difficult to assess, because both pre- and post-menopausal patients, as well as patients using LH–RH analog participated in this study. Second, because we did not evaluate direct anti-tumor effects such as clinical response or recurrence, we cannot rule out the possibility that even patients with relatively low exposure to the active metabolites (such as endoxifen levels < 15 ng/ml) could have received similar clinical benefits with enough exposure. Similarly, exposure achieved with TOR 120 mg may provide no advantage over that with TOR 40 mg in terms of clinical efficacy, because there are limited data for a direct comparison of the two doses. Third, since we could not conduct comprehensive analysis of all possible variant genotypes, this might have classified a few patients into different category. Fourth, total activity calculated in this study was not widely used. Because analysis for TOR has not been well conducted in the past, it was difficult to select single metabolite for estimation of its activity, since all metabolites contribute to the activity. Fifth, PK sampling time in this study might not be optimal and can be a cause of variability. Finally, although we have excluded the patients who regularly use CYP2D6 inhibitor, such as paroxetine, use of all currently established strong or moderate CYP2D6 inhibitors was not excluded.

A Cochrane meta-analysis of seven randomized-controlled trials indicated that TOR and TAM are equally effective in patients with advanced breast cancer, although some toxicities such as headache (risk ratio 0.14, *p* = 0.02), ocular disorders (risk ratio 0.46, *p* = 0.10), and endometrial cancer (risk ratio 0.22, *p* = 0.10) seem to be more common for TAM [15]. This analysis included mostly Western patient populations (*n* = 2061), except for one Japanese study with only 57 patients in each arm. The prevalence of the *CYP2D6* allele with reduced activity (CYP2D6*10) is significantly higher in Asian populations [16]. Therefore, we need to be careful when adapting clinical trial data obtained from mostly Caucasian patients, because they have significantly different genetic backgrounds to Asian patients.

In a genotype-guided intra-patient dose-escalation study of TAM conducted in the US, the endoxifen concentrations among patients homozygous for *CYP2D6 PM* (11/118 patients) genotype (*3, *4, *5, *6) were still significantly lower (less than one-half of the endoxifen concentrations for EMs) despite receiving a double dose (40 mg) of TAM [10]. A genotype-guided intra-patient dose-escalation study of TAM conducted in Japan also suggested that patients homozygous for CYP2D6*10 may achieve lower concentrations of endoxifen despite receiving 40 mg of TAM [6]. Because the contribution of CYP2D6 to TOR metabolism is relatively small, TOR might be a valid alternative to TAM, especially in patients predicted to be PMs of TAM. Further clinical trials are warranted to validate this concept.

Although the concentrations of the 4OH metabolites of TAM, such as endoxifen, in relation to the *CYP2D6* genotype and TAM dose have been measured in clinical studies in both Western and Asian populations [6, 10], the active metabolites of TOR have not been determined in clinical trial samples. The results of a recent in vitro study suggested that the contribution of CYP2D6 to the bioactivation of TOR is lower than that of TAM [11].

In conclusion, genotyping for *CYP2D6* is not enough to predict endoxifen or the total activity of TAM, because there are large overlaps between different genotype groups; therefore, a strategy incorporating therapeutic drug monitoring may be more logical, especially in populations with higher proportions of PMs, such as Asian populations. Furthermore, for patients found to be PMs of TAM, TOR may be a good candidate; however, this approach needs to be validated in prospective clinical trials using efficacy as an endpoint.

## Electronic supplementary material

Below is the link to the electronic supplementary material.

Supplementary material 1 (DOCX 17 KB)

Supplementary material 2 (TIFF 3999 KB)
