# Supplementary material for: Pharmacogenomic–pharmacokinetic study of selective estrogen-receptor modulators with intra-patient dose escalation in breast cancer
Source: Breast Cancer. 2019 Feb 7;26(5):535–43. doi: 10.1007/s12282-019-00952-9 (PMC6694038; doi:10.1007/s12282-019-00952-9)
Supplement: Supplementary file 1 — Supplementary material 1 (DOCX 17 KB) [file 12282_2019_952_MOESM1_ESM.docx]

**Title**

Pharmacogenomic–pharmacokinetic study of selective estrogen-receptor modulators with intra-patient dose escalation in breast cancer

**Journal Name**

Breast Cancer

**Corresponding author**

Prof. Hiroshi Ishiguro

Department of Medical Oncology, International University of Health and Welfare Hospital

E-mail: [hishiguro@iuhw.ac.jp](mailto:hishiguro@iuhw.ac.jp)

Table S1. Summary of the concentrations of TAM, TOR, and their metabolites

|  | Dose of TOR | Mean | SD | Median | Min | Max | N |
| --- | --- | --- | --- | --- | --- | --- | --- |
| TOR (ng/mL) | 40 mg | 735.7 | 205.5 | 764.0 | 321.0 | 1300.0 | 61 |
| NDM-TOR (ng/mL) | 40 mg | 1373.7 | 390.8 | 1350.0 | 601.0 | 2820.0 | 61 |
| 4OH-TOR (ng/mL) | 40 mg | 8.1 | 3.2 | 6.9 | 2.4 | 16.4 | 61 |
| 4OH-NDM-TOR (ng/mL) | 40 mg | 15.4 | 8.5 | 13.4 | 4.5 | 68.8 | 61 |
| Total activity (ng/mL) | 40 mg | 3984.8 | 1343.7 | 3730.2 | 1614.5 | 9827.0 | 61 |
| TOR (ng/mL) | 120 mg | 1957.5 | 643.1 | 1890.0 | 992.0 | 3330.0 | 27 |
| NDM-TOR (ng/mL) | 120 mg | 3594.4 | 1218.0 | 3500.0 | 1380.0 | 5430.0 | 27 |
| 4OH-TOR (ng/mL) | 120 mg | 16.0 | 6.4 | 14.6 | 5.5 | 31.2 | 27 |
| 4OH-NDM-TOR (ng/mL) | 120 mg | 32.6 | 11.6 | 34.3 | 11.5 | 52.9 | 27 |
| Total activity (ng/mL) | 120 mg | 9247.4 | 2922.4 | 9343.3 | 3781.5 | 14911.3 | 27 |
| TAM (ng/mL) | - | 174.7 | 67.4 | 163.0 | 38.5 | 552.0 | 182 |
| NDM-TAM (ng/mL) | - | 283.2 | 122.8 | 271.0 | 59.2 | 1090.0 | 182 |
| 4OH-TAM (ng/mL) | - | 3.6 | 1.6 | 3.3 | 0.2 | 9.5 | 182 |
| Endoxifen (ng/mL) | - | 23.5 | 12.0 | 22.8 | 1.8 | 64.5 | 182 |
| Total activity (ng/mL) | - | 1871.2 | 768.9 | 1762.9 | 186.5 | 4569.3 | 182 |

Table S2. Adverse events in the intra-patient dose-escalation clinical pharmacology study for TOR (n = 14)

|  |  | 40 mg | 120 mg  (1) | 120 mg  (2) | p for 40 mg and 120 mg (1) | p for 40 mg and 120 mg (2) | p for 120 mg (1) and (2) |
| --- | --- | --- | --- | --- | --- | --- | --- |
| Weight (kg) | Mean | 53.2 | 53.4 | 53.6 | 0.34 | 0.08 | 0.03 |
|  | SD | 7.3 | 7.4 | 7.4 |  |  |  |
|  | Median | 51.7 | 51.0 | 51.1 |  |  |  |
|  | Min | 45.0 | 45.0 | 46.0 |  |  |  |
|  | Max | 73.4 | 73.5 | 73.0 |  |  |  |
|  | Missing | 1 | 1 | 3 |  |  |  |
| Hot flush | G0 (%) | 14 (100) | 9 (69.2) | 9 (75.0) | - | - | 1.00 |
|  | G1 (%) | 0 (0) | 2 (15.4) | 1 (8.3) |  |  |  |
|  | G2 (%) | 0 (0) | 2 (15.4) | 2 (16.7) |  |  |  |
|  | Missing | 0 (0) | 1 | 2 |  |  |  |
| GOT elevation | All grade (%) | 0 (0) | 2 (16.7) | 1 (8.3) | - | - | - |
|  | ≧ G2 (%) | 0 (0) | 1 (8.3) | 0 (0) |  |  |  |
|  | Missing | 2 | 2 | 2 |  |  |  |
| GPT elevation | All grade (%) | 0 (0) | 1 (8.3) | 1 (8.3) | - | - | 1.00 |
|  | ≧ G2 (%) | 0 (0) | 1 (8.3) | 0 (0) |  |  |  |
|  | Missing | 2 | 2 | 2 |  |  |  |
| T. Bil  elevation | All grade (%) | 0 (0) | 0 (0) | 0 (0) | - | - | - |
|  | ≧ G2 (%) | 0 (0) | 0 (0) | 0 (0) |  |  |  |
|  | Missing | 4 | 3 | 2 |  |  |  |
| Cre elevation | All grade (%) | 4 (36.4) | 3 (27.3) | 2 (16.7) | 1.0 | 1.0 | 1.0 |
|  | ≧ G2 (%) | 0 (0) | 0 (0) | 0 (0) |  |  |  |
|  | Missing | 3 | 3 | 2 |  |  |  |
| LDL elevation | All grade (%) | 0 (0) | 1 (14.3) | 1 (10.0) | - | - | 1.0 |
|  | ≧ G2 (%) | 0 (0) | 0 (0) | 0 (0) |  |  |  |
|  | Missing | 7 | 7 | 4 |  |  |  |
| TG elevation | All grade (%) | 1 (12.5) | 0 (0) | 0 (0) |  |  |  |
|  | ≧ G2 (%) | 0 (0) | 0 (0) | 0 (0) |  |  |  |
|  | Missing | 6 | 5 | 2 |  |  |  |
| Low WBC | All grade (%) | 1 (11.1) | 1 (11.1) | 1 (8.3) | 1.0 | 1.0 | 1.0 |
|  | ≧ G2 (%) | 1 (11.1) | 1 (11.1) | 1 (8.3) |  |  |  |
|  | Missing | 5 | 5 | 2 |  |  |  |
| Low PLT | All grade (%) | 0 (0) | 0 (0) | 0 (0) |  |  |  |
|  | ≧ G2 (%) | 0 (0) | 0 (0) | 0 (0) |  |  |  |
|  | Missing | 5 | 5 | 2 |  |  |  |
| LDH elevation | All grade (%) | 0 (0) | 1 (7.1) | 0 (0) |  |  |  |
|  | ≧ G2 (%) | 0 (0) | 1 (7.1) | 0 (0) |  |  |  |
|  | Missing | 0 | 0 | 0 |  |  |  |
| Hyperglycemia | All grade (%) | 0 (0) | 0 (0) | 1 (7.1) |  |  |  |
|  | ≧ G2 (%) | 0 (0) | 0 (0) | 1 (7.1) |  |  |  |
|  | Missing | 0 | 0 | 0 |  |  |  |

A paired t-test or McNemer’s tests for comparison between 49 mg and 120 mg (2)

p-value not calculated in case of no events
