# Supplementary figures and images for: Pharmacogenomic–pharmacokinetic study of selective estrogen-receptor modulators with intra-patient dose escalation in breast cancer
Source: Breast Cancer. 2019 Feb 7;26(5):535–43. doi: 10.1007/s12282-019-00952-9 (PMC6694038; doi:10.1007/s12282-019-00952-9)

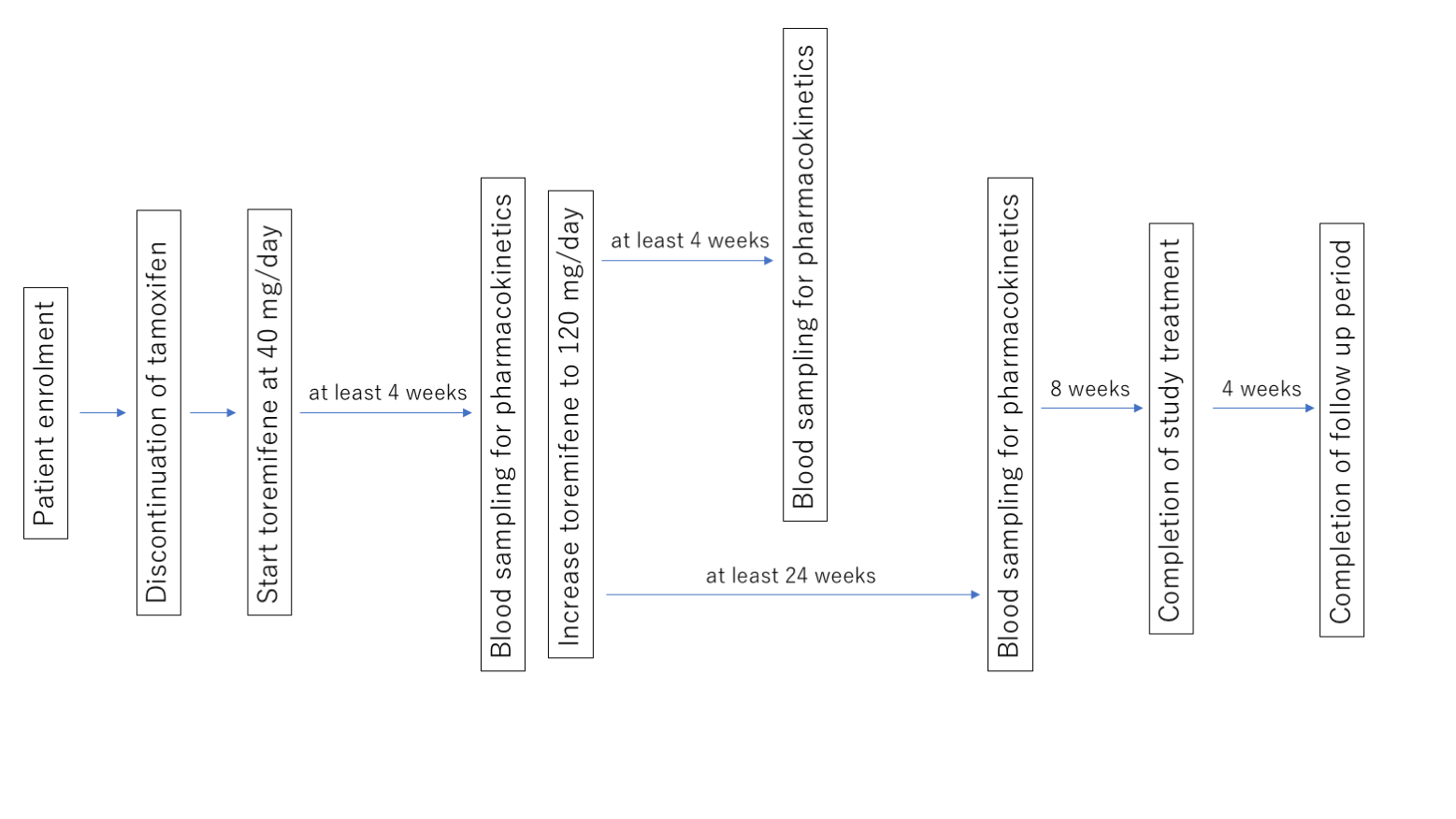

Supplement: Supplementary file 2 — Supplementary material 2 (TIFF 3999 KB) [file 12282_2019_952_MOESM2_ESM.tiff]
